# TET Enzymes and 5hmC in Adaptive and Innate Immune Systems

**DOI:** 10.3389/fimmu.2019.00210

**Published:** 2019-02-12

**Authors:** Chan-Wang J. Lio, Anjana Rao

**Affiliations:** ^1^Division of Signaling and Gene Expression, La Jolla Institute, La Jolla, CA, United States; ^2^Department of Pharmacology and Moores Cancer Center, University of California, San Diego, La Jolla, CA, United States; ^3^Sanford Consortium for Regenerative Medicine, San Diego, CA, United States

**Keywords:** 5hmC, 5 hydroxymethylcytosine, ten eleven translocation (TET), DNA modification, epigenetics (methylation/demethylation), gene regulation and expression

## Abstract

DNA methylation is an abundant and stable epigenetic modification that allows inheritance of information from parental to daughter cells. At active genomic regions, DNA methylation can be reversed by TET (Ten-eleven translocation) enzymes, which are responsible for fine-tuning methylation patterns. TET enzymes oxidize the methyl group of 5-methylcytosine (5mC) to yield 5-hydroxymethylcytosine (5hmC) and other oxidized methylcytosines, facilitating both passive and active demethylation. Increasing evidence has demonstrated the essential functions of TET enzymes in regulating gene expression, promoting cell differentiation, and suppressing tumor formation. In this review, we will focus on recent discoveries of the functions of TET enzymes in the development and function of lymphoid and myeloid cells. How TET activity can be modulated by metabolites, including vitamin C and 2-hydroxyglutarate, and its potential application in shaping the course of immune response will be discussed.

## Introduction

Cells rely on the proper propagation and preservation of epigenetic information in order to regulate gene expression appropriately. 5-methylcytosine (5mC), described as the 5th base of DNA, is a chemically stable modification that is one of the most reliable ways of transmitting epigenetic information. In most cells, 5mC is present primarily at symmetrically-methylated CG dinucleotides in DNA, although methylation of cytosines in other contexts (CH=CA, CT, CC) has been reported in stem cells and in neurons (1). During DNA replication, methylated CGs are replaced by unmodified cytosines in the newly synthesized DNA strand, and the resulting hemimethylated CGs are recognized by a complex of UHRF1 and the maintenance methyl-transferase DNMT1 (2–4). The remethylation of hemi-methylated CpGs in newly replicated DNA is complete within 20 min, accounting for the stable inheritance of DNA methylation (5). In contrast to DNMT1, which depends on 5mC deposition at CpG motifs for maintenance DNA methylation, the *de novo* methyltransferases DNMT3A and DNMT3B can methylate unmodified cytosines in both CG and CH sequence contexts. While the writers for DNA methylation (DNMTs) have been known for decades, how DNA methylation is removed remained unclear until the discovery of TET (Ten-Eleven Translocation) enzymes and their ability to oxidize 5mC to 5-hydroxymethyl-cytosine (5hmC) [(6); reviewed in (3, 4)].

5hmC, the so-called 6th base, is a stable epigenetic modification that accounts for 1–10% of 5mC depending on the cell type: ~10% in embryonic stem cells (6) and as high as 40% in Purkinje neurons (7). While 5hmC or related modifications have been known to exist in simpler organisms including T-even phages for more than half a century (8), it was not until 2009 that 5hmC was rediscovered in mammalian cells (6, 7). The mammalian enzymes responsible for generating this modification are the three TET dioxygenases (TET1, TET2, and TET3) that utilize the co-factors α-ketoglutarate (αKG), reduced iron (Fe^2+^), and molecular oxygen to oxidize the methyl group at the 5 position of 5mC (6). TET proteins can be found in every metazoan organism that contains DNMTs, even simple organisms such as comb jellies (9–11).

**Figure 1:**
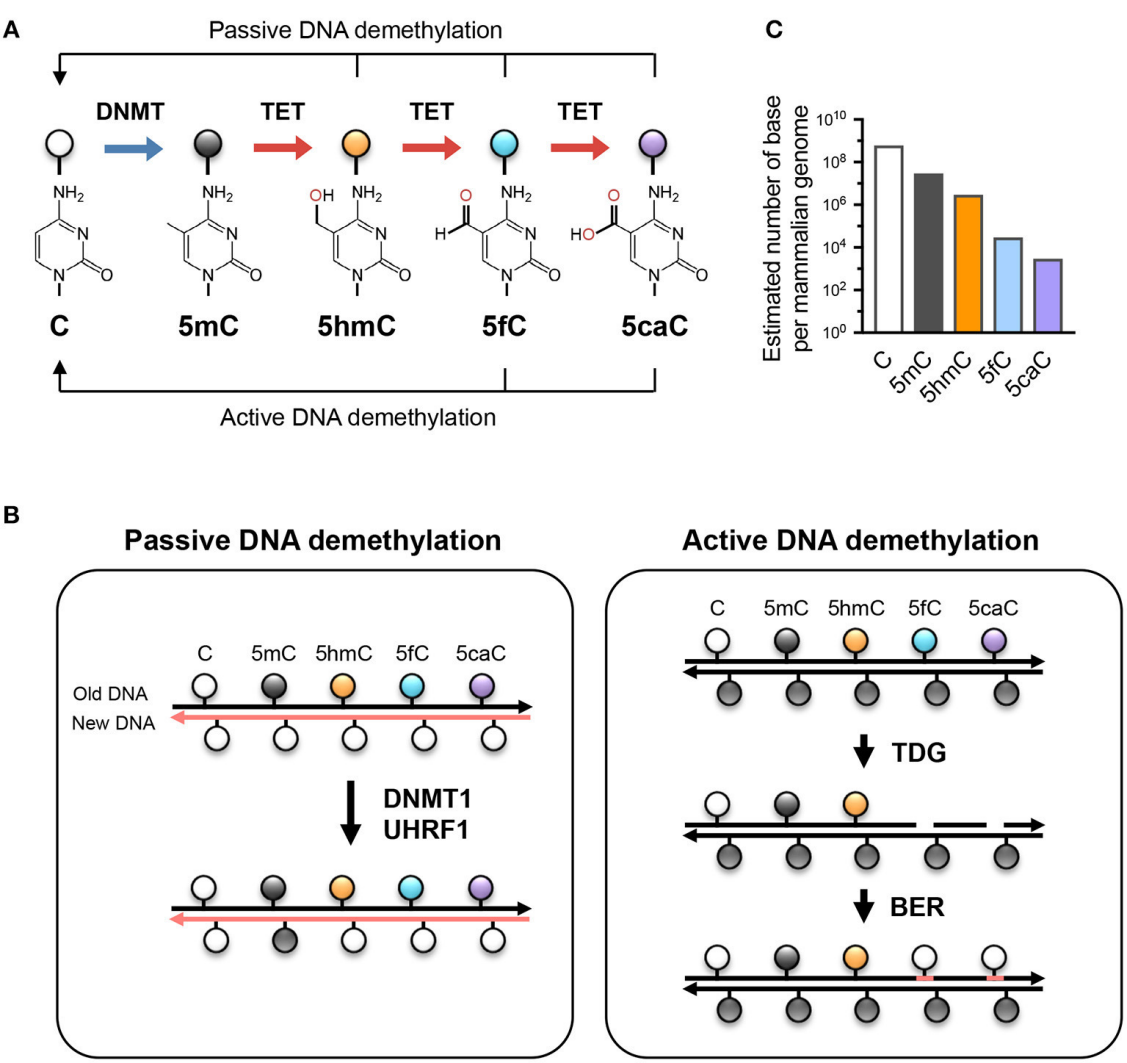
TET-mediated DNA modifications and demethylation. **(A)** Unmodified cytosine (C) is methylated by DNA methyltransferases (DNMTs) at the 5 position to become 5-methylcytosine (5mC). TET proteins oxidize 5mC into 5-hydroxymethylcytosine (5hmC), a stable epigenetic mark, and subsequently to 5-formylcytosine (5fC) and 5-carboxylcytosine (5caC). TET can demethylate DNA via replication-dependent (passive) or replication-independent (active) mechanisms. **(B)** Left, passive DNA demethylation. DNMT1/UHRF1 complex recognizes 5mC at the hemi-methylated CpG motif during DNA replication and methylates the unmodified cytosine on the newly synthesized DNA strand (left; pink strand). However, the oxidized methylcytosines 5hmC, 5fC, and 5caC (together, oxi-mC) are not recognized by DNMT1/UHRF1, resulting in unmodified cytosine on the new DNA strand. Further DNA replication in the presence of continuing TET activity will result in progressive dilution of 5mC in the daughter cells. *Right panel*, active DNA demethylation. While 5hmC is stable and persists in the genome, 5fC and 5caC can be recognized and excised by thymine DNA glycosylase (TDG), and the resulting abasic sites are repaired as unmodified C by base excision repair (BER). Other mechanisms (e.g., decarboxylation of 5caC) have been suggested but have not yet been proven to exist. **(C)** The approximate abundance of unmodified and modified cytosines in the haploid human/mouse genome. About 5% of cytosine is methylated (5mC); in most cells, the vast majority of 5mC is present at CG dinucleotides although it is low at CpG islands. 5hmC amounts to about 1-10% of 5mC (estimated at 10% here as in embryonic stem cells), while the levels of 5fC and 5caC are each about an order of magnitude lower than the previous oxidative modification. It is not known whether the low levels of 5fC and 5caC are due to features of TET enzymes that cause them to arrest at 5hmC, or to their continuing removal by TDG or other mechanisms.

Besides being a potential epigenetic mark, 5hmC is the key intermediate for TET-mediated active (replication-independent) and passive (replication-dependent) DNA demethylation (Figure 1). TET enzymes iteratively oxidize 5mC and 5hmC into other oxidized cytosines (oxi-mCs) including 5-formylcytosine (5fC) and 5-carboxylcytosine (5caC) (12); in active DNA demethylation, 5fC and 5caC are recognized and excised by thymine DNA glycosylase (TDG), repaired by the base-excision repair system, and replaced by unmodified C, thus resulting in DNA demethylation (13). In replication-dependent passive DNA demethylation, the DNMT1/UHRF1 complex does not recognize hemi-modified CGs with 5hmC, 5fC, or 5caC and thus the cytosine on the newly synthesized DNA strand is not methylated (5, 14, 15). Thus, the interplay between DNMT and TET proteins sculpts the DNA methylation landscape and enables the flow of epigenetic information across cell generations.

**Figure 2:**
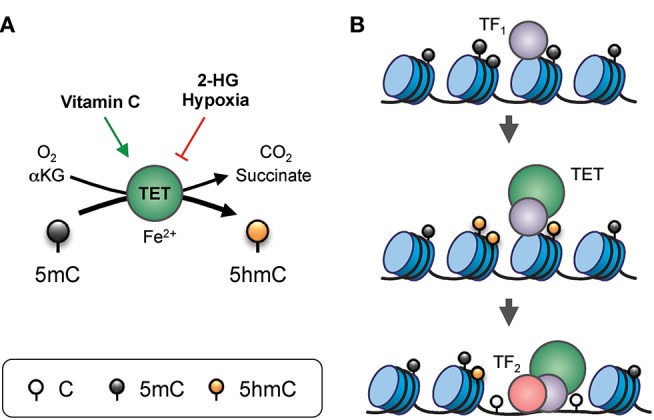
Gene regulation by TET proteins. **(A)** Enzymatic activity of TET. TET proteins, with the co-factors Fe^2+^ and α-ketoglutarate (αKG), use oxygen to oxidize 5mC into 5hmC, generating CO_2_ and succinate as by-products. The enzymatic activity of TET can be modulated by additional factors. For instance, vitamin C (ascorbate) can enhance TET activity, potentially via reduction of the iron ion. On the other hand, the “oncometabolite” 2-hydroxyglutarate (2-HG), generated in acute myeloid leukemia and glioblastoma by recurrent dominant-active mutants of isocitrate dehydrogenase 1 or 2 (IDH1/2), inhibits TET activity. Furthermore, lack of oxygen in hypoxia also inhibits TET function. **(B)** Model of TET-mediated enhancer regulation. Prior to the commissioning of an enhancer, pioneer transcription factor (indicated as TF_1_) binds to nucleosomal DNA and recruits TET which oxidizes the surrounding 5mC into 5hmC (and/or other oxi-mCs), facilitating DNA demethylation. TET proteins and, TF_1_ promote enhancer accessibility by recruiting nucleosome remodeling complexes, thus allowing binding of secondary transcription factors (indicated here as TF_2_) that are otherwise inhibited by DNA methylation or the presence of nucleosomes.

DNA modification by TET proteins is essential for gene regulation (Figure 2). TET3 is expressed in the oocyte and the zygote; all three TET proteins are expressed in blastocysts; TET1 and TET2 are expressed in embryonic stem (ES) cells; and TET2 and TET3 are expressed ubiquitously in differentiated cells (3, 4). The three TET enzymes appear to have overlapping but distinct targets in the genome. For instance, in mouse ES cells, TET2 rather than TET1 is responsible for the vast majority of 5hmC generation, and TET1 preferentially facilitates promoter demethylation while TET2 and TET3 act on enhancers (16, 17). The longstanding association of high-level gene transcription with low levels of promoter methylation may be explained by TET-mediated conversion of 5mC to 5hmC at promoters, and subsequent DNA demethylation.

The genome-wide distribution of 5hmC reflects the strong association of TET enzymes with gene transcription. 5hmC is enriched at the most active enhancers and the gene bodies of the most highly transcribed genes (18). Moreover, multiple transcription factors important in cell differentiation and lineage specification, including NANOG, SALL4A, WT1, EBF1, PU.1, and E2A, have been shown to recruit TET proteins to specific genomic loci (primarily enhancers) for 5hmC modification, in most cases marking them for subsequent demethylation (19–24). As a result, TET function is particularly essential for gene transcription during cell activation and lineage specification, and deficiencies of TET protein expression or activity result in skewed or arrested cell differentiation in multiple lineages, including those in neural and hematopoietic systems (25–30).

**Figure 3:**
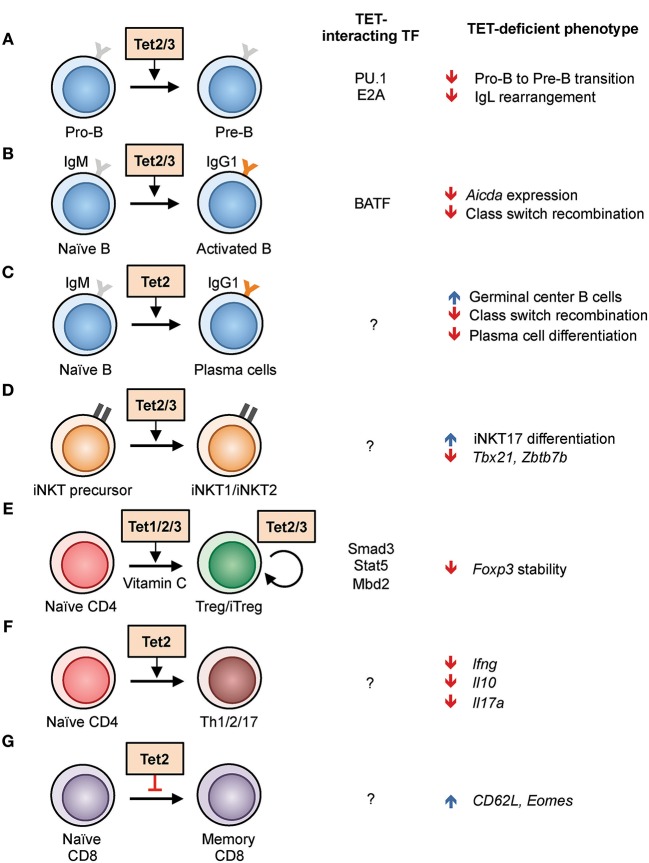
Regulation of lymphoid development and function by TET proteins in the mouse. **(A–G)** List of known TET functions in lymphoid cells. The interacting transcription factors and the phenotypes found in *TET*-deficient mice are shown in the right columns. **(A)** The use of *Mb1-cre Tet2/3*-deficient mice showed that *Tet2* and *Tet3* regulate the pro-B to pre-B cell transition, in part by enhancing the rearrangement of immunoglobulin light chains (22, 37). **(B)** Acute deletion of *Tet2/3* using *Cre*^*ERT*2^ in B cells resulted in decreased *Aicda* expression and thus class switch recombination (28). **(C)** Deletion of *Tet2* using *Vav-Cre* and *Cd19-Cre* resulted in hyperplasia of germinal center B cells. *Vav-Cre*-driven *Tet2* deletion resulted in decreased plasma cell differentiation (38). **(D)** *Cd4-cre Tet2/3*-deficient mice exhibited skewed differentiation toward iNKT17 cells, partly due to decreased expression of *Tbx21* and *Zbtb7b* expression, and a massive T-cell-receptor-dependent expansion of affected T cells (33). **(E)** Tet proteins facilitate the *in vitro* differentiation of naïve CD4 T cells to iTreg cells by demethylating *Foxp3* enhancer *CNS2*, a process enhanced by the presence of vitamin C. All three TET proteins have a role in stabilizing the expression of *Foxp3* in Treg cells *in vivo* (39, 40). **(F)** CD4 T cells from *Cd2-cre Tet2*-deficient mice showed impaired Th1, Th2, and Th17 differentiation and cytokine production (41). **(G)** Increased differentiation of CD8 memory cells from *Cd4-cre Tet2*-deficient mice in response to lymphocytic choriomeningitis virus infection (42).

**Figure 4:**
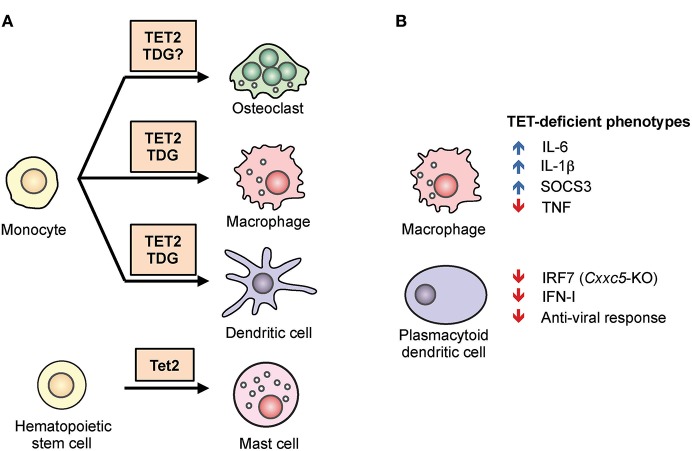
The role of TET2 in myeloid differentiation and function. **(A)** TET2 regulates myeloid cell differentiation. TET2, together with the thymine DNA glycosylase (TDG), facilitates active DNA demethylation and promotes lineage-specific gene expression during the differentiation of osteoclasts, macrophages, and dendritic cells from human monocytes. In mice, TET2 is required for the differentiation of mast cells *in vitro* and *in vivo* (43, 44). **(B)** TET2 regulates the function of myeloid cells. In mouse and human macrophages, TET2 repressed expression of the pro-inflammatory cytokines IL-1β and IL-6 (45–48). TET2 was shown to associate with Iκbζ, bind to the *Il6* promoter, recruit HDAC2, and repress *Il6* expression (46). *Tet2*-deficient macrophages also expressed a high level of *Socs3* mRNA, which in normal mice was suggested to be demethylated by TET2 and subsequently degraded by ADAR1 (44). In plasmacytoid dendritic cells, CXXC5 recruited TET2 to an intragenic CpG island in *Irf7*, facilitating the demethylation and maintaining basal expression. As a result, loss of *Cxxc5*, and to a lesser extent *Tet2*, resulted in decreased levels of IRF7, decreased type I interferon (IFN-I) production, and decreased anti-viral responses (49).

TET loss-of-function is strongly connected to oncogenesis (31, 32). Especially in the hematopoietic system, arrested or skewed cell differentiation is often associated with cell transformation (22, 26). In humans, *TET2* is one of the most frequently mutated genes in hematopoietic cancers of both myeloid and lymphoid origin (26). Using mouse models, we and other groups have shown that deletion of *Tet2* alone, or deletion of both *Tet2* and *Tet3* (the two TET enzymes with the greatest overlap in expression and function), leads to myeloid or lymphoid expansion and the development of aggressive cancers with 100% penetrance (22, 25, 33). For instance, a striking example is the inducible deletion of both *Tet2* and *Tet3* in adult mice, which leads to acute myeloid leukemia with the mice succumbing as early as 3 weeks post-deletion (25). Since the role of TET proteins in malignancies has been reviewed extensively (26, 34–36), we will focus here on their roles in immune cell development and function. In the sections below, we outline our current understanding of the roles of TET proteins in regulating the adaptive and innate immune systems. The major findings are summarized in Figures 3, 4.

## TET Proteins in T and B Cell Differentiation and Function

During development and immune responses, T and B cells continuously receive signals from antigen and cytokine receptors. These external signals converge and are interpreted by combinations of ubiquitously expressed and cell type-specific transcription factors, which function together with chromatin regulators to remodel the epigenome. The epigenetic changes associated with immune cell activation and differentiation include DNA and histone modifications, which allow information to be stored and/or inherited by daughter cells. As noted above, analyses of genome-wide 5hmC distribution reveal a close relationship between 5hmC and gene transcription. In thymic and peripheral T cell subsets, the level of 5hmC at gene bodies shows a striking positive correlation with the level of gene expression, as well as occupancy by RNA polymerase II and the level of H3K36me3 histone modification, an epigenetic mark reflective of RNA transcription into the gene body (17, 18, 50, 51). Similarly, in lymphoid cells, 5hmC showed a strong positive correlation with enhancer activity, denoted by the level of H3K27 acetylation (H3K27ac), suggesting that TET is important for regulating enhancer function (16, 18, 52). Indeed, recent studies of T and B cells from our lab (see below) and others demonstrated that one of the functions of TET proteins is to facilitate chromatin accessibility at enhancers (22, 28, 33, 37). TET-mediated conversion of 5mC to 5hmC potentially disrupts the binding of 5mC-binding proteins including MeCP2 and MBD (Methyl-CpG-binding domain) proteins, facilitating nucleosome remodeling and the binding of transcription factors (53, 54). These changes in the epigenetic status of enhancers are likely transmitted to daughter cells, thus facilitating the establishment of lineage identity.

### TET Proteins in B Cell Development and Function

From bone marrow progenitors to peripheral memory and plasma cells, the B cell genome undergoes progressive demethylation following differentiation (55). Furthermore, *TET2* is one of the most frequently mutated genes (6–12%) in diffuse large B cell lymphoma, a malignancy originating from germinal center B cells (56–58). These observations suggest that TET proteins play an important role in B cell biology. Indeed, when *Tet2* and *Tet3* were deleted in early B cells during bone marrow development using *Mb1-Cre*, B cell differentiation was arrested at the transition from the pro-B to the pre-B stage (22, 37) (Figure 3A). One function of these TET proteins during early B cell development is to regulate the arrangement of the Ig kappa (Igκ) light chain genes, that pairs with the rearranged Ig heavy chain to form the complete B cell receptor. TET proteins regulate Igκ rearrangement by oxidizing 5mC at Igκ enhancers and facilitating their DNA demethylation and chromatin accessibility.

Mechanistically, TET proteins appear to be recruited to enhancers by “pioneer” transcription factors, defined by their ability to associate with their binding motifs on nucleosome-bound DNA. Our data indicate that in pro-B cells, the pioneer transcription factor PU.1 binds to the Igκ enhancers prior to light chain rearrangement as a placeholder and recruits Tet proteins for DNA demethylation, facilitating the binding of additional B cell transcription factors including E2A (Figure 3A). Tet proteins also regulate the expression of IRF4 and IRF8, both of which are important for Igκ rearrangement (22). Similar to the expansion phenotype observed in T cells (described in more detail below), *Mb1-Cre Tet2/3*-deficient mice developed massive expansion of immature B cells resembling acute lymphoblastic lymphoma (22). Therefore, Tet proteins are essential for B cell development by controlling the expression of multiple key genes.

In mature B cells, TET proteins are important for the antibody response. Recently we showed that B cell activation induced Tet protein expression and changes in the genome-wide distribution of 5hmC (the hydroxymethylome): lipopolysaccharide (LPS) and IL-4 stimulation induced a progressive TET-dependent hydroxymethylation at ~8,000 regions in the span of 3 days (28). Functionally, the two major members in naïve B cells TET2 and TET3 are crucial for antibody class switch recombination (CSR) (Figure 3B). Acute deletion of *Tet2* and *Tet3* by *Cre*^*ERT*2^ resulted in an ~50% decrease the expression of AID (Activation Induced Deaminase; encoded by *Aicda*), the critical enzyme for CSR; reconstitution of catalytically active AID in *Tet2/3*-deficient B cells restored CSR. Interestingly, the CSR phenotype is reminiscent of that resulting from *Aicda* haploinsufficiency (59, 60), suggesting that TET proteins are required for optimal expression of *Aicda*. Mechanistically, we showed that the transcription factor BATF recruits Tet proteins to the *Aicda* superenhancer, facilitating hydroxymethylation and chromatin accessibility of two *Tet*-responsive elements, *TetE1* and *TetE2*, within the superenhancer and augmenting the expression of *Aicda* (28) (Figure 3B).

Recently, *Vav-Cre* and *Cd19-Cre*, which are expressed in the entire hematopoietic system and during B cell development, respectively, were used to show that disruption of *Tet2* resulted in germinal center hyperplasia (38) (Figure 3C). However, germinal center B cells appeared to be normal in *Tet2* deletion driven by *C*γ*1*-Cre, which is expressed in germinal center B cells. Consistent with our findings, *Tet2* was shown to be required for CSR and affinity maturation of antibody (Figure 3C). More importantly, TET2 positively regulated the expression of the transcription factor *Prdm1* (encoding BLIMP1), and plasma cell differentiation was impaired in *Tet2*-deficient mice. Interestingly, the gene signature of *TET2*-deficient DLBCL resembles that of cells with mutations in the histone acetyltransferase *CREBBP*, suggesting that TET2 and CREBBP may cooperate to regulate enhancer H3K27 acetylation. Taken together, these observations demonstrate that TET proteins regulate multiple processes in B cells by preferentially strengthening the activity of enhancers, including individual enhancer elements located within super-enhancers (Igκ and *Aicda*) (61).

### TET Proteins in T Cell Development

*Tet2* and *Tet3* are expressed at higher levels than *Tet1* in thymocytes and peripheral T cells, and are responsible for the majority of 5hmC modification in these cells. Deletion of *Tet2* alone in the germline, in the hematopoietic system using *Cd2-cre*, or in T cells (*Cd4-cre*) did not lead to any obvious defect in T cell development (41, 42, 62), suggesting that *Tet3* was able to compensate for the loss of *Tet2*. Indeed, data from our lab showed that the deletion of both *Tet2* and *Tet3* in T cells using *Cd4-cre* caused a massive lymphoproliferative phenotype with enlarged spleen and lymph nodes, and the mice succumbed by 8 weeks of age (33). At 3–4 weeks of age, young *Tet2/3 Cd4-cre DKO* mice showed decreased thymic cellularity, a lower percentage of CD4^+^CD8^+^ double positive cells, and an increased percentage of CD4^+^ and CD8^+^ single positive cells, phenotypes reminiscent of thymic atrophy induced by stress or inflammation. Further examination showed that the expanded cells in the periphery were invariant natural killer T (iNKT or NKT) cells that expressed the transcription factor Rorγt and produced IL-17 (Figure 3D). These cells thus resemble the NKT17 subset, one of the three subsets of NKT cells besides NKT1 (T-bet-expressing) and NKT2 (Gata3-expressing). In contrast, NKT cells from wildtype mice are primarily of the NKT1 and NKT2 subsets (63).

Genome-wide analyses provided mechanistic explanations for the lineage skewing observed in *Tet2/3 Cd4-cre DKO* mice. Briefly, the profiles of transcriptome, whole-genome methylome, and chromatin accessibility showed that *Tet2/3* deficiency resulted in decreased expression of *Tbx21* (encoding T-bet) and *Zbtb7b* (encoding Th-POK), likely because of hypermethylation at the corresponding regulatory elements (Figure 3D). Both T-bet and Th-POK repress *Rorc* (encoding Rorγt) expression, thus the decreased levels of T-bet and Th-POK transcription factors in *Tet2/3*-deficient cells permitted increased Rorγt expression and skewed the cells to the NKT17 lineage (33). Interestingly, the *Tet2/3*-deficient iNKT cells were able to expand upon transfer to fully immunocompetent, wild-type (WT) but not *Cd1d*-deficient recipient mice (33), suggesting (i) that the expansion was secondary to recognition of “self” antigens presented by CD1d and (ii) that expansion was not suppressible by WT regulatory T (Treg) cells (see below). Together, these observations indicate that TET enzymes are important to maintain the proper expression of lineage-specifying transcription factors, and to limit the differentiation and proliferation of overly self-reactive cells including iNKT cells.

### Maintenance of Foxp3^+^ Treg Cells Requires TET Proteins

TET enzymes are important for the homeostasis of T regulatory (Treg) cells, which are distinguished from other T cell lineages by their expression of the transcription factor FOXP3. In Treg cells, TET2 and TET3 are required for stable *Foxp3* expression through their ability to demethylate two intronic enhancers, termed conserved non-coding sequence (*CNS*) 1 and *CNS2* (39, 64) (Figure 3E). Bisulfite sequencing showed that the *Foxp3 CNS1* and *CNS2* enhancers were hypermethylated in Treg cells from *Tet2/3 Cd4-cre DKO* mice (39). Moreover, overexpression of the TET1 catalytic domain in CD4 cells induced to differentiate into *Foxp3*-expressing induced Treg cells (iTreg) *in vitro* and resulted in partial demethylation of *CNS2* (65), suggesting that TET enzymes may be in constant balance with the methylation machinery. Hypermethylation at *Foxp3 CNS2* was also observed in *Tet1/2*-deficient mice, suggesting all three Tet proteins may function redundantly in regulating *Foxp3* (40).

Several proteins have been identified to partner with TET proteins in regulating *Foxp3 CNS2*. For instance, loss of the DNA methyl-binding protein MBD2 also resulted in hypermethylation of *CNS2* (also termed TSDR), potentially because of decreased TET2 binding (66). How MBD2 cooperates with TET to demethylate *CNS2* remained to be determined. Besides MBD2, the transcription factors SMAD3 and STAT5, induced by TGFβ and IL-2 respectively, recruit TET proteins to *Foxp3 CNS2* and facilitate DNA demethylation (40). In addition, the level of TCR and cytokine stimulation has been linked to the degree of DNA demethylation at *Foxp3 CNS2* (67). Since there is only one functional allele of *Foxp3* per Treg cell, this observation implies that stronger a TCR stimulation might increase the probability of TET-mediated DNA demethylation at *Foxp3 CNS2* and concomitantly, the stability of *Foxp3* expression.

### TET Proteins Link Metabolism to Foxp3 Expression

The enzymatic activity of TET can be influenced by various factors, including the level of co-factors αKG, oxygen, and vitamin C (Figure 2). In a chemical screen using mouse embryonic stem cells, vitamin C was found to enhance the expression of gene expression in germ cells and ES cells by facilitating TET-mediated DNA demethylation at their promoters (68). Vitamin C treatment also facilitated TET-mediated demethylation of *Foxp3 CNS2* and stabilized *Foxp3* expression in differentiating induced Treg (iTreg) cells (Figure 3E). Inhibition of the vitamin C transporter by sulfinpyrazone confirmed the role of vitamin C and TET proteins in *CNS2* demethylation and the generation of peripheral Treg cells *in vivo* (69). In addition, vitamin C facilitated the conversion of mouse and human naïve CD4 T cells into iTreg cells induced by TGFβ and retinoic acid with improved stability and suppressive function (39). Besides vitamin C, another metabolite hydrogen sulfide (H_2_S) was shown to be required for Treg cell differentiation, at least in part by increasing *Tet1* and *Tet2* expression (40).

TET activity can be inhibited by the “oncometabolite” 2-hydroxyglutarate (2-HG), a competitive inhibitor of αKG-dependent dioxygenases including TET (70, 71) (Figure 2). 2-HG is a normal metabolite that exists as two stereoisomers, R-2-HG and S-2-HG; the latter is considerably more potent at inhibiting TET activity (72). In the past few years, it has become clear that 2-HG can be generated via multiple pathways; for instance, recurrent mutations of isocitrate dehydrogenase 1 and 2 (IDH1/2) give rise to dominant-active enzymes with the novel property of converting isocitrate to the R enantiomer of 2-HG (R-2-HG) (70, 71). A recent study identified a compound, (aminooxy)acetic acid (AOA), that is able to reprogram differentiating Th17 cells into *Foxp3*-expressing iTreg cells (73). Metabolic profiling identified the target of AOA in Th17 cells as GOT1 (glutamate oxaloacetate transaminase 1), an enzyme that catalyzes the conversion of glutamate to αKG. Th17 cells express a high level of GOT1 compared to iTreg cells, consistent with their elevated level of αKG. However, instead of facilitating the function of TET enzymes and other dioxygenases, the αKG is converted by wild-type IDH1/2 into R-2-HG, which inhibits TET activity, promotes increased methylation at *Foxp3 CNS2*, and represses *Foxp3* expression. By targeting GOT1, the small molecule AOA effectively decreased the intracellular level of R-2-HG and allowed TET proteins to demethylate *CNS2*, favoring differentiation to iTreg cells at the expense of the Th17 lineage (73). Therefore, these observations suggest that, besides conveying signals from cell surface receptors, TET proteins also integrate environmental cues into the epigenome.

### TET Proteins Regulate Peripheral T Cell Differentiation and Function

After stimulation and depending on the extracellular signal received, naïve CD4 T cells can differentiate into multiple lineages, including Th1, Th2, Th17, follicular T helper cells (Tfh), and Treg. Analysis of 5hmC distribution in peripheral T cells showed a positive correlation between gene expression level and 5hmC modification at gene bodies, including those of the lineage-specific transcription factor *Tbx21* and *Gata3* for Th1 and Th2 cells, respectively. This observation suggests that TET proteins may regulate the differentiation of peripheral T cells (18, 41). Similar lineage-specific 5hmC modifications during Th1 and Th2 polarization were also reported in human CD4 T cells (74). Indeed, *Tet2*-deficient murine CD4 T cells produced less IFNγ and IL-17 when polarized *in vitro* to Th1 and Th17, respectively (41) (Figure 3F). Compared to WT cells, adoptively transferred *Tet2*-deficient CD4 T cells were more pathogenic in an experimental autoimmune encephalomyelitis (EAE) model, and immunization with myelin oligodendrocyte glycoprotein (MOG) peptide induced significantly less IFNγ and IL-10 but a similar level of IL-17 (41). These observations reinforce the idea that Tet proteins are important for proper lineage differentiation and gene expression.

Analysis of *Tet2*-deficient (*Tet2*^*fl*/*fl*^ *Cd4-cre*) CD8 T cells responding to infection with lymphocytic choriomeningitis virus (LCMV) showed increased LCMV gp33-specific memory precursor cells (KLRG1^−^ CD127^+^) and decreased short-lived effector cells (KLRG1^+^ CD127^−^) on day 8 post-infection (42) (Figure 3G). These memory-like cells expressed CD27, CD62L, and CXCR3, a phenotype similar to central memory cells, and persisted for at least 45 days post-infection with a higher level of Eomes compared to WT. Transfer of *Tet2*-deficient memory cells conferred better protection against gp33-expressing *Listeria monocytogenes* compared to WT memory cells, strongly suggesting that TET2 represses memory cell differentiation (42). In addition to TCR-induced TET protein expression (42), TET activity can also be modulated by physiologically produced 2-HG. CD8 T cells generate substantial levels of the potent 2-HG enantiomer “oncometabolite” S-2-HG as early as day two after TCR stimulation, coinciding with the decrease in 5hmC (75). Similar to genetic ablation of *Tet2*, S-2-HG treatment of CD8 T cells induced higher expression of Eomes and CD62L, markers for central memory cells. Surprisingly, OT-I CD8 T cells cultured in the presence of S-2-HG *in vitro* displayed enhanced survival and tumor clearance upon adoptive transfer *in vivo* (75), suggesting the effect of S-2-HG is long lasting by stably remodeling the epigenome.

In humans, TET loss-of-function was shown to have a major potentiating role in a case of cancer immunotherapy against B cell malignancy using T cells bearing the anti-CD19 chimeric antigen receptor (CAR). The patient bore a hypomorphic mutation in one allele of *TET2*, and the CAR lentivirus serendipitously became integrated into the other *TET2* allele. The resulting profound loss of function of *TET2* resulted in an almost monoclonal expansion of this particular CAR-T cell, and the patient went into complete remission (76). Thus the loss of *TET2* activity resulting from insertional mutation of one *TET2* allele due to lentiviral integration, combined with the preexisting hypomorphic mutation in the other *TET2* allele, led to superior anti-tumor function and again conferred a central memory phenotype on the expanded CAR-T cells. Together, these observations show that TET proteins are important in regulating peripheral T cell differentiation.

## TET Proteins in Myeloid Differentiation and Function

### TET2 Regulates Myeloid Differentiation

*TET2* mutation has been closely linked to myeloid malignancies including myelodysplastic syndrome and acute myeloid leukemia in human (26). In mice, germline disruption of the *Tet2* gene decreased the global level of 5hmC in hematopoietic stem cells (HSCs), enhanced HSC survival and proliferation, inhibited T, B, and erythroid differentiation, and biased differentiation toward the myeloid lineage (62). Similarly, knockdown of *TET2* in human cord blood CD34^+^ progenitor cells decreased total 5hmC in the cells and skewed their differentiation toward the granulomonocytic lineage, specifically monocytes, at the expense of both lymphoid and erythroid lineages (77). These and other studies suggested that, compared to other lineages, the myeloid lineage requires less reconfiguration of the DNA methylome during differentiation and therefore is relatively unaffected in the absence of TET2.

Beyond HSC, TET2 also regulates the differentiation of mast cells (Figure 4A). In a model of *in vitro* mast cell differentiation in which bone marrow progenitors were cultured with IL-3, loss of *Tet2* inhibited mast cell differentiation, decreased cytokine production, and induced aberrant hyperproliferation (43). Two major transcription factors involved in myeloid development, C/EBPα and C/EBPε, were up-regulated in *Tet2*-deficient mast cells, and both contributed to the observed defect in differentiation. Similar to another observation in macrophages (see below), both catalytically active and inactive TET2 could partially rescue these phenotypic defects, suggesting that part of the function of TET2 is to maintain the structure of a repressive protein complex (43). *In vivo, Tet2* is important for the expansion of mast cells induced by parasites (44).

Human monocytes can differentiate into macrophages (MACs), dendritic cells (DCs), and osteoclasts (OCs) *in vitro* depending on cytokine signals, and the epigenetic regulation of this process has been studied extensively (78). During post-mitotic differentiation of DCs from monocytes, stimulation with cytokines GM-CSF and IL-4 induced DNA demethylation. Since these cells do not proliferate prior to differentiation, the mechanism of demethylation is assumed to involve an active replication-independent process. Similar observations were made during MAC and OC differentiation (20, 79–81). TET2-mediated oxidation of 5mC into 5hmC preceded and was required for DNA demethylation, which was accompanied by the presence of active histone modifications (H3K4me1, H3K4me3, H3/H4 acetylation) (Figure 4A). In general, the degree of DNA demethylation at distal elements or promoters showed a loose positive correlation with gene expression with numerous exceptions, suggesting that additional mechanisms contributed to gene regulation, such as H3K27 methylation by the polycomb complex (82). In monocyte to DC differentiation, IL-4-activated STAT6 induced TET2-dependent demethylation, and this was important for acquiring the proper cell identity and priming the expression of inducible genes (e.g., *IL1B, CCL20*) (81). During monocyte to OC or to MAC differentiation, the transcription factor PU.1 was found to associate with both hypo- and hypermethylated regions and to directly bind to TET2 as well as to the DNA methyltransferase DNMT3B (20). TET2 functioned together with thymine-DNA glycosylase (TDG), and to a lesser extent with activation-induced deaminase (AID), to hydroxymethylate and demethylate DNA, facilitating the establishment of cell-type-specific gene expression programs (83). The same study also showed that TET2 was responsible for recruiting the histone H3K4 methyltransferase SETD1A, and for increasing H3K4me3 modification at cell-type specific genes examined (83).

Together, these *in vitro* human studies showed that post-mitotic myeloid cells utilize TET2 and TDG for replication-independent, active DNA demethylation to establish cell-specific gene expression patterns or to prime gene for subsequent induction. Besides regulating lymphoid development, Tet proteins are required for the differentiation of multiple lineages of myeloid cells.

### TET Proteins Regulate Immune Responses by Myeloid Cells

One function of TET proteins in normal myeloid cells appears to be the repression of inflammatory gene expression (Figure 4B). For instance, *Tet2*-deficient macrophages and dendritic cells expressed a higher level of IL-6 in response to stimulation (45, 46). Mechanistically, TET2 was shown to associate with Iκbζ and bind to the *Il6* promoter, recruiting the histone deacetylase HDAC2 and repressing *Il6* expression. As discussed for mast cells above, the repression appeared to be independent of TET2 catalytic activity, suggesting that TET2 provided a structural scaffold for the formation of a repressive complex (46). Compared to WT controls, *Tet2*-deficient mice were more susceptible to endotoxin-induced septic shock and dextran sulfate sodium (DSS)-induced colitis, coincident with an increased IL-6 level (46).

TET proteins also repressed another inflammatory cytokine, IL-1β (47, 48). Moreover, loss of *Tet2* accelerated atherosclerosis development in a mouse model of low-density lipoprotein receptor (*Ldrl*) deficiency (47). *Tet2*-deficient macrophages increased IL-1β secretion via the NLRP3 inflammasome, the inhibition of which protects mice from atherosclerosis (47). Interestingly, IL-1R/MyD88 signaling was shown to induce Tet2 mRNA and protein expression in bone marrow-derived macrophages (84), suggesting a potential negative feedback loop controlling IL-1β expression by TET proteins. Lastly, TET2 facilitated immunosuppression by tumor-infiltrating myeloid cells in a melanoma model and loss of TET2 in myeloid cells inhibited melanoma growth *in vivo* (84), consistent with the role of TET proteins in suppressing inflammation in myeloid cells. TET proteins contribute to osteoclast differentiation and suppress inflammation, and osteoclast activation has been linked to rheumatoid arthritis (RA) (85), warranting further detailed investigation of the role of TET proteins in autoimmune and auto-inflammatory diseases.

TET proteins appear to have different functions in myeloid cells depending on the circumstances. For instance, TET proteins have been reported to promote myeloid immune responses and production of inflammatory cytokines rather than suppressing inflammation. Plasmacytoid dendritic cells (pDCs) are fast responders to infection and are able to produce a large quantity of type I interferon. This ability has previously been attributed to their high basal level of the transcription factor IRF7, the expression of which is regulated by an intronic CpG island (CGI) (49). TET2 is recruited to this locus by the zinc-finger protein CXXC5, and is required to maintain the demethylated status of the CGI (Figure 4B). As a result, mice deficient in *Cxxc5*, or to a lesser extent *Tet2*, were more vulnerable to infection by herpes simplex virus and vesicular stomatitis virus due to an impaired interferon response (49). Similarly, in a model of abdominal sepsis, *Tet2* deficiency was shown to reduce infection-induced myelopoiesis with a decreased level of TNFα and chemokines (44). The authors suggested that instead of oxidizing DNA, TET2 repressed *Socs3* expression by oxidizing methylcytosine in the 3′ untranslated region of *Socs3* RNA, thereby facilitating ADAR1-mediated destabilization of the mRNA in a manner independent of the normal RNA-editing function of ADAR1 (44) (Figure 4B). Although TET proteins are capable of oxidizing methylcytosine on RNA (86, 87), whether TETs can demethylate RNA (i.e., replace 5mC with unmodified C) is still an open question as neither passive nor active mechanisms for DNA demethylation would apply in RNA (Figure 1).

Finally, it is worth noting that the phenotypes in *Tet2*-deficient mice may be complicated due to environmental influences. Whole-body *Tet2* deficiency was shown to result in a compromised intestinal barrier, allowing bacteria to translocate from the intestinal lumen to internal organs and induce IL-6 production and inflammation; in turn, the pro-inflammatory signal facilitated pre-leukemic myeloproliferation (88). Therefore, depending on the microbiota at a given facility, *Tet2*-deficient mice may display differing basal levels of inflammation, a feature that may account for the variable reported phenotypes of different strains of *Tet2*-deficient mice (26). Since most *TET2* mutations in human are acquired somatically rather than through the germline, the extent to which inflammation plays a role in human myeloid neoplasms remains to be determined. Taken together, these studies provide clear evidence that TET proteins regulate innate immune responses in myeloid cells.

## Our Current Understanding of TET-Mediated Gene Regulation

### TET Regulation of Transcription Factor Expression in Immune System

Transcription factors have emerged as one of the major targets of TET-mediated regulation. For instance, TET2 is important for inducing *Blimp1* expression in peripheral B cells by demethylating intronic CpGs (38). On the other hand, TET proteins may be required for repressing *BCL6* expression. In the human *BCL6* locus, DNA methylation of intragenic CpG islands at the first intron prevents CTCF binding and promotes *BCL6* expression. DNA demethylation at these CpG islands allowed CTCF binding, resulting in repressed *BCL6* expression (89). However, whether TET proteins regulate *BCL6* expression remains to be demonstrated.

Many loci encoding transcription factors are heavily hydroxymethylated, including *Tbx21, Zbtb7b*, and *Gata3* in iNKT and T cells (18, 33, 41). Loss of *Tet2* alone, however, has no significant effect on *Tbx21* expression in CD4 and CD8 T cells (41, 42). It is likely that other TET proteins such as TET3 can compensate, since *Tbx21* expression is decreased in iNKT cells that are deficient in both *Tet2* and *Tet3* (33). In contrast to *Tbx21* which is decreased in TET-deficient iNKT cells, loss of TET activity, either by gene targeting or inhibition by 2-HG, facilitates *Eomes* expression in iNKT and CD8 T cells (33, 42, 75). Whether TET proteins directly regulate *Tbx21* and *Eomes* expression by binding to regulatory elements in the *Tbx21* and *Eomes* loci remains to be determined.

### TET-Mediated Regulation of Enhancers

Consistent with the functions of TET proteins in gene regulation, enhancers are usually enriched in 5hmC. TET proteins can be recruited to specific regulatory elements through interaction with multiple transcription factors including NANOG, SALL4A, WT-1, PU.1, E2A, and EBF1 (19–24). The pleiotropic interaction between TET proteins and transcription factors is reminiscent of histone acetyltransferase p300, which interacts with hundreds of transcription factors (90). Once recruited to enhancers, TET proteins can oxidize 5mC into 5hmC, marking enhancers for DNA demethylation.

TET-dependent DNA modifications potentially affect gene expression via at least two non-mutually exclusive mechanisms. First, 5hmC, other oxi-mCs, and the ensuing DNA demethylation increase chromatin accessibility (22, 28, 33). In this scenario, unmodified C and oxi-mC potentially relieve the nucleosome rigidity caused by DNA methylation (91, 92); additionally, TET proteins may recruit nucleosome remodeling complexes to displace nucleosomes from enhancers. Second, TET-generated oxi-mC modifications may exert immediate effects on gene expression by modulating transcription factor binding, and TET proteins may also exert more long-term effects. Specifically, 5mC and oxi-mCs are known to modify the binding of several transcription factors with CG or TG dinucleotides in their recognition sequences (54). The methyl group of thymine is located at the 5th position, corresponding to the methyl group of 5mC. Thus, transcription factors with TG dinucleotides in their preferred binding sequences often also bind the same sequences with methylated CGs (93), and their DNA binding is likely to be modified by the presence of oxi-mCs. Other transcription factors, including WT1, can bind sequences containing 5caC in a CG context with higher affinity than the corresponding sequence with unmodified CG (94). The exact mechanisms of enhancer regulation by TET enzymes and oxi-mCs remain to be delineated.

### TET-Mediated DNA Oxidation and Demethylation

TET proteins can oxidize 5mC into oxi-mCs and mediate DNA demethylation. Depending on the conditions, TET2 can iteratively oxidize 5mC to 5hmC and then to all other oxidized cytosines in a single encounter (95). However, in the genome, most 5mC oxidation appears to pause at 5hmC and to a lesser extent 5fC (Figure 1C), a notion supported by mass spectrometric analyses showing that both 5hmC and 5fC are rather stable in cells (96, 97). It remains to be determined why 5hmC is the most abundant of the oxi-mCs. Two mechanisms (not mutually exclusive) may be involved: (i) TET-mediated oxidation preferentially arrests at 5hmC or 5fC; (ii) 5fC and 5caC, but not 5hmC, are continuously removed by TDG/ BER or by other mechanisms (Figure 1B). Regardless of the mechanism, the modified cytosines can facilitate active or passive demethylation and affect gene regulation. In addition, 5hmC may act as a bookmark to label CpG sites in *cis*-elements such as promoters, enhancers and insulators marked by CTCF binding (5, 98, 99) for subsequent demethylation upon cell division, thus affecting gene expression patterns in the daughter cells (a latent effect).

### Potential Co-transcriptional 5hmC Modification

5hmC distribution at gene bodies is positively correlated with gene expression levels, suggesting that TET activity is coupled to transcription by RNA polymerase II (RNA pol II) (18). One of the possible links between TET and RNA pol II is via their mutual association with the histone H3K4 methyltransferase Set1/COMPASS complex (100). Another possible link between 5hmC and RNA transcription is via the gene body histone mark H3K36me3: the levels of 5hmC and H3K36me3 in gene bodies are positively correlated with one another and with gene expression. During transcription, the methyltransferase SETD2 associates with the phosphorylated C-terminal domain of RNA pol II and co-transcriptionally methylates H3K36 to yield H3K36me3 (94). H3K36me3 is subsequently recognized by the *de novo* DNA methyltransferases DNMT3B, and to a lesser extent DNMT3A, via the PWWP domain (101–103), mediating gene body DNA methylation. Since all three TET proteins have been shown to co-immunoprecipitate with the maintenance methyltransferase DNMT1, and all three DNMT proteins co-immunoprecipitate with TET2 (104), the extensive interaction between TET and DNMT may provide a possible mechanism for transcription-coupled 5hmC modification. The biological significance of gene body 5hmC modification remains to be determined.

### Potential Model for TET-Mediated Asymmetric Cell-Fate Decision

Hypothetically, it may also be possible to facilitate asymmetric gene regulation by engineering an asymmetric distribution of DNA methylation between two daughter cells via strand-biased 5hmC modifications. In one potential scenario, 5mC bases at CpG motifs on one strand at a given locus are preferentially oxidized by TET into 5hmC, while the complementary strand remains as 5mC (e.g., the template strand during transcription). As a result, after cell division, the CpG motifs at the locus in one of the daughter cell will remain methylated because the DNMT1/UHRF1 complex restores symmetrical methylation; the CpG motifs in the other daughter cell will contain 5hmC and unmodified C. This is an attractive putative mechanism by which TET enzymes could regulate cell fate decisions.

## Harnessing the Power of the Dark Side for the Light Side

TET loss-of-function, either through genetic mutations or catalytic inhibition, has shown a strong causal relationship with multiple malignancies (31, 32). TET deficiency appears to enhance cell survival and increase “stemness,” as in the case of *TET*-deficient HSCs which could be passaged for a much longer period of time *in vitro* and out-competed WT HSCs after transplantation *in vivo*. Interestingly, at least some of the phenotypes are reversible by re-introducing TET or enhancing the remaining TET activity by vitamin C (105), raising the possibility of temporarily inhibiting TET activity to enhance immune responses. In fact, two recent studies of human and mouse CD8 T cells provided supporting evidence for this approach. In both cases, *TET2*-deficiency facilitated the differentiation and expansion of CD8 T cells with central memory phenotype that could provide long-lasting protection against tumor and virus (discussed above). Using non-specific inhibitors such as the oncometabolite 2-HG or other TET-specific inhibitors that remain to be developed, it should be possible to inhibit TET activity and boost antigen-specific responses and immune cell expansion during vaccination or infusion of cancer-specific T cells. It would be of great interest to borrow the trick of losing TET function from cancer cells to arm immune cells with the superpower to fight against the cancer cells themselves and other pathogens.

## Concluding Remarks

TET proteins and 5hmC were identified/rediscovered almost 10 years ago. Numerous studies have shown their importance in gene regulation, tumor suppression, and cell differentiation. Yet, much remains to be learned about TET and 5hmC. For instance, how do TET enzymes suppress cancer progression? How does TET-mediated DNA modification affect cell identity? What is the relative contribution of enzymatic activity-dependent and –independent (structural) mechanisms to the functions of TET? Besides being intermediates for DNA demethylation, what is the function of 5hmC and other oxidized methylcytosines as potential epigenetic marks? Who are the “readers” of these epigenetic marks? Also, given their seemingly opposite functions, why do mutations of *Tet* and *Dnmt3a/b* result in similar phenotypes in hematopoiesis? Besides all these fundamental questions, modulating the activity of epigenetic regulating enzymes including TET proteins may provide a promising way to alter and to achieve the desired magnitude and direction of immune responses.

## Author Contributions

All authors listed have made a substantial, direct and intellectual contribution to the work, and approved it for publication.

### Conflict of Interest Statement

AR is a member of the Scientific Advisory Board of Cambridge Epigenetix. The remaining author declares that the research was conducted in the absence of any commercial or financial relationships that could be construed as a potential conflict of interest.
